# Eliminating Medicaid dental benefits and early‐stage oral cancer diagnoses

**DOI:** 10.1002/cam4.7061

**Published:** 2024-03-08

**Authors:** Jason Semprini, Julie Reynolds, Whitney E. Zahnd, George Wehby

**Affiliations:** ^1^ Department of Health Management and Policy University of Iowa College of Public Health Iowa City Iowa USA

**Keywords:** dental, Medicaid, oral cancer, oropharyngeal, policy, SEER

## Abstract

**Background:**

Despite the importance of regular dental visits for detecting oral cancer, millions of low‐income adults lack access to dental services. In July 2009, California eliminated adult Medicaid dental benefits. We tested if this impacted oral cancer detection for Medicaid enrollees.

**Methods:**

We analyzed Surveillance, Epidemiology, and End Results‐Medicaid data, which contains verified Medicaid enrollment status, to estimate a difference‐in‐differences model. Our design compares the change in early‐stage (Stages 0–II) diagnoses before and after dropping dental benefits in California with the change in early‐stage diagnoses among eight states that did not change Medicaid dental benefits. Patients were grouped by oropharyngeal cancer (OPC) and non‐OPC (oral cavity cancer), type, and the length of Medicaid enrollment. We also assessed if the effect of dropping dental benefits varied by the number of dentists per capita.

**Results:**

Dropping Medicaid dental benefits was associated with a 6.5%‐point decline in early‐stage diagnoses of non‐OPC (95% CI = −14.5, −3.2, *p* = 0.008). This represented a 20% relative reduction from baseline rates. The effect was highest among beneficiaries with 3 months of continuous Medicaid enrollment prior to diagnosis who resided in counties with more dentists per capita. Specifically, dropping dental coverage was associated with a 1.25%‐point decline in the probability of early‐stage non‐OPC diagnoses for every additional dentist per 5000 population (*p* = 0.006).

**Conclusions:**

Eliminating Medicaid dental benefits negatively impacted early detection of cancers of the oral cavity. Continued volatility of Medicaid dental coverage and provider shortages may be further delaying oral cancer diagnoses. Alternative approaches are needed to prevent advanced stage OPC.

## Graphical abstract

In July 2009, California eliminated their Medicaid dental benefits. Using novel Surveillance, Epidemiology, and End Results‐Medicaid cancer registry data, we found that this policy change reduced early‐stage diagnoses of non‐oropharyngeal oral cancer for Medicaid enrollees.

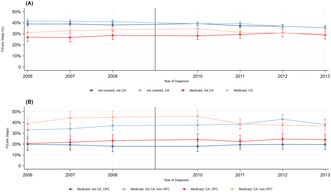

## 1 INTRODUCTION

Dentists are often on the frontlines of oral cancer detection and care.^1^ Given that earlier diagnosis is critical for patient survival and quality of life, dentists play an essential role in oral cancer detection.^2^, ^3^, ^4^ Dental professionals conduct over 75% of all oral cancer screenings.^5^ They are also the only health care provider in the United States to systematically screen for oral cancers during routine examinations.^6^, ^7^ However, less than 30% of oral cancers were diagnosed early in 2019.^8^ Patients who are diagnosed with early‐stage (Stages 0–II) oral cancer likely visit a dental prior to diagnosis.^1^ Dental professionals also contribute to prevention by assisting adult patients with smoking or alcohol cessation and encouraging adolescent patients to receive cancer prevention vaccines.^9^, ^10^, ^11^ For oral cancer patients receiving radiation therapy, regular dental treatment can treat infections, ease eating or speaking discomfort, and prevent further damage to the oral cavity.^12^, ^13^, ^14^

Despite the importance of regular dental visits for detecting oral cancer, millions of low‐income adults lack access to dental services.^15^ More adults delay dental care due to cost than any other health care service.^15^ Medicaid provides access to health care for millions of people. However, many states limit or restrict Medicaid dental benefits for adults.^16^, ^17^ Even among adults with dental coverage, or the ability to pay out of pocket, access barriers remain. Sixty million adults live in counties designated as dental professional shortage areas; requiring an influx of over ten thousand new dental practitioners to address the unmet need.^18^ Moreover, not all dentists accept insurance, and fewer yet accept patients enrolled in Medicaid.^19^, ^20^, ^21^ Public policies improving the affordability and availability of dental care remain warranted. Addressing the volatility of Medicaid dental systems is most urgent for low‐income adults, who bear the greatest burden of oral cancer amidst a widening gap in dental service utilization trends among poor and nonpoor adults.^15^, ^22^

### 1.1 Eliminating Medicaid dental benefits in California

California eliminated adult Medicaid dental benefits in 2009, causing over three million low‐income adults to lose their dental coverage.^23^ With the exception of pregnant women and nursing home residents, the policy change eliminated all nonemergency dental benefits for Medicaid enrollees.^24^ While comprehensive dental coverage returned in 2014, the elimination of dental benefits had clear negative effects on patients' access to dental care over the previous 5 years.^17^ Dental visits for low‐income adults dropped by 20%.^25^ Without Medicaid dental benefits, enrollees shifted their dental care to higher cost emergency departments.^26^, ^27^ Younger adults and racial minority groups in urban areas reported the largest increase in emergency dental claims.^26^, ^27^ People with disabilities also experienced a decrease in dental utilization.^25^

The elimination of Medicaid dental benefits also reduced the availability of dental care at public clinics, which, prior to the cuts, received revenue for services provided to Medicaid covered low‐income adults, the primary patient population at these clinics.^24^ In rural areas and communities with high poverty, the number of practicing dentists declined 10%–20%.^24^

### 1.2 Medicaid dental benefits and oral cancer detection

The potential consequences of dropping Medicaid dental coverage for low‐income adults at risk of developing oral cancer could be dire. Medicaid dental coverage and benefits impact Medicaid dental care.^28^ Among the services most responsive to changing dental benefits are comprehensive exams: the service where most oral cancer screenings are performed.^5^, ^29^ By hindering access to comprehensive exams, dropping Medicaid dental benefits may have slowed the detection of oral cancer among low‐income adults. However, low‐income adults have among the lowest rates of self‐reported oral cancer screening.^5^ Therefore, it is not clear if and by how much reduced access to comprehensive dental exams would negatively impact oral cancer detection among Medicaid enrollees. Our objective was to examine the effect of dropping Medicaid dental benefits on stage at diagnosis for oral cancers. Specifically, the study employs a quasi‐experimental model to newly released cancer registry dataset with verified Medicaid enrollment indicators to evaluate how California's 2009 policy to eliminate Medicaid dental benefits impacted early‐stage oral cancer diagnoses in the low‐income adult population. Additionally, our study evaluates whether the policy effects varied by availability of dental professionals and length of Medicaid enrollment.

## 2 METHODS

### 2.1 Research design

We estimated the association between dropping Medicaid dental benefits in California in 2009 and the probability of an early‐stage oral diagnosis with a difference‐in‐differences design.^30^ This approach accounts for temporal trends and unobserved confounding contemporaneous with the state's policy change. The design compares changes in the probability of early‐stage diagnoses for California before and after the elimination of dental benefits to the changes in the other states that did not experience such a policy change, and subtracts the changes between California and the control states.

### 2.2 Data and sample selection

We used the National Cancer Institute's Surveillance, Epidemiology, and End Results (SEER) program data.^31^ This newly released, restricted SEER‐Medicaid file links SEER cancer case data to verified date of Medicaid enrollment indicators for the years 2006–2013.^32^ For the first time, this SEER cancer data report if the patient was enrolled in Medicaid at the time of diagnosis, as well as 3 months leading up to the diagnosis. For this study, the analytical sample included adults diagnosed with oral and pharynx cancer and enrolled in Medicaid in the month of diagnosis. In alternate samples, we only included those with Medicaid coverage in the month or 2 months prior to diagnosis (irrespective of coverage at diagnosis) or those 3 months of continuous Medicaid enrollment at the time of diagnosis to account for interruption in coverage around diagnosis. As a falsification check, we separately evaluated the policy for oral cancer patients who were not enrolled in Medicaid at any time during the three months leading to the oral cancer diagnosis.

Cancer cases were restricted to tumors of the “Oral Cancer and Pharynx” based on ICD‐O‐3/WHO 2008 classification codes.^33^ Given the heterogeneity by tumor type, the sample was also stratified to separately analyze oropharyngeal cancers (OPC): base of tongue, tonsil, oropharynx, and pharynx; and non‐oropharyngeal cancer (non‐OPC) or cancers of the oral cavity: lips, anterior tongue, gingivae, floor of mouth, palate, and other oral cavities.^34^ Stage at diagnosis was defined using Derived AJCC Stage Group, 6th Ed (2004–2015) Collaborative Staging.^35^ The outcome was being diagnosed at an early‐stage (Stages 0–II), compared to an advanced stage (Stages III and IV).

Deidentified patient data on sociodemographic covariates were age, sex, race/ethnicity, and marital status. The sample was further restricted to adults with a confirmed age of 19–99. Dual Medicare‐Medicaid eligible adults were included in the sample. Despite their reliance on Medicaid for accessing dental services (Medicare provides no Medicaid dental benefit), dual‐eligible enrollees were not exempt from the loss of adult Medicaid dental benefits.^24^

The SEER‐Medicaid data file includes state‐county geocodes and encrypted census tract‐level socioeconomic indices. These census‐tract “Yost” quintiles, which rank the socioeconomic status (SES) of each census‐tract within a state, are included as covariates.^36^ County‐level Rural‐Urban Continuum Codes were added to code for metropolitan status. County‐level data on the number of dentists per capita were obtained from the Agency for Healthcare Research and Quality.^37^

### 2.3 Exposure

Individuals were the unit of analysis and assigned to the policy treatment if they resided in California after the state eliminated dental benefits (July 2009 to December 2013). All other states are considered controls as these states never changed their Medicaid dental benefits during the study period (Connecticut, Georgia, Iowa, Kentucky, Louisiana, Michigan, New Jersey, and New Mexico).^17^ We excluded cancer cases from Hawaii, Utah, and Washington from our analysis because they changed their Medicaid dental program during the study period.^17^, ^38^

### 2.4 Statistical analysis

With the goal of estimating the association between dropping Medicaid dental benefits and the probability of an early‐stage oral cancer diagnosis, we constructed the following regression model:

(1) $$P\left(Y_{\text{iecst}}\right|\text{Oral Cancer})=f\left(B*{\text{CALIFORNIA}}_{s}*{\text{POST}}_{t}+X_{\text{icst}}+\text{METR}O_{c}+\mathrm{SE}S_{e}+\text{MONT}H_{m}+\mathrm{YEA}R_{t}+\text{STAT}E_{s}\right).$$

In Equation 1, *B* is the parameter of interest and estimates the effect of dropping Medicaid dental benefits on the probability of oral cancer diagnoses. CALIFORNIA_s_ is a (time‐invariant) indicator for patients residing in California versus other states. POST_t_ is an indicator for diagnosis after California drops dental benefits (≥2009) compared to before. Note, California eliminated all nonemergency adult dental benefits effective July 1, 2009.^24^ *Y*_icts_ is the binary indicator for an early stage of oral cancer diagnosis for individual i, in county c, at year t, in state *s*. *X*_icst_ includes sex, race/ethnicity, age, and tumor site for each individual i in census tract e in county c, month m, state s, and year t. METRO_c_ is a binary indicator for living in a metro versus nonmetro county. SES_e_ is a vector of binary variables corresponding to the individual's census‐tract level “Yost” SES quintile. Month_m_ includes binary indicators for month of diagnosis to account for any seasonal variation in oral cancer diagnoses. YEAR_t_ is a set of binary variables for year of diagnosis to capture secular trends or shocks affecting oral cancer detection patterns and shared across states. STATE_s_ is a set of binary variables for state of residence which control for unobserved, time‐invariant heterogeneity affecting oral cancer detection differently between states. Because we include YEAR_t_ and STATE_s_ directly as covariates, there is no need to include CALIFRONIA_s_ and POST_t_ separately as covariates other than in the interaction term (they would be perfectly collinear).

To test if the association of dropping Medicaid dental coverage varies by dentist supply, measures of available dental professionals, Equation 1 is augmented by a county‐level, time‐invariant variable DENTISTS_c_ and the number of dentists per 5000 population.^18^, ^37^

(2) $$\begin{array}{c} P\left(Y_{\text{iecst}}\right|\text{Oral Cancer})\\ =f\left(a*{\text{CALIFORNIA}}_{s}*{\text{POST}}_{t}*\text{DENTIST}S_{c}+X_{\text{icst}}+\text{METR}O_{c}+\mathrm{SE}S_{e}+\text{MONT}H_{m}+\mathrm{YEA}R_{t}+\text{STAT}E_{s}\right). \end{array}$$

This specification, akin to a triple difference model, estimates whether effect of exposure to dropping Medicaid dental coverage in state s at year t varies by the number of dentists in the county (captured by parameter a). A priori, we expect the effect of removing dental benefits to be more pronounced in areas with more dentists per capita as prior research has shown that benefits of Medicaid dental insurance expansions on utilization are more pronounced with greater availability of dentists.^29^, ^39^

All models were estimated by a linear probability regression model. Standard errors were clustered at the state level.^40^ To account for the small number of states (clusters) and that only one is the treatment state, standard errors were also derived using Wild Cluster Bootstrap.^41^ This approach alleviates issues with small number of clusters to produce valid 95% confidence interval coverage.^41^, ^42^ Significance levels are set at *a* = 0.05. To further examine the validity of our estimates, we construct a set of event‐history studies to test for pretreatment differential trends in oral cancer diagnoses between states, before California dropped Medicaid dental benefits.^43^

## 3 RESULTS

The analytical sample included 7552–9596 individuals enrolled in Medicaid depending on the length or timing of enrollment at diagnosis and the OPC‐status of the tumor. The placebo sample included 50,013 were not enrolled in Medicaid during the study period. Table 1 reports the baseline sample descriptive statistics for the sample of Medicaid enrollees.

**TABLE 1:** Descriptive statistics of adult Medicaid beneficiaries diagnosed with oral cancer (SEER‐Medicaid: 2006–2008).

	Not enrolled in Medicaid		Enrolled in Medicaid at diagnosis	
	Not California	California	Not California	California
	*N* (%)	*N* (%)	*N* (%)	*N* (%)
Early stage (0–II)	4648 (38.5%)	3896 (41.2%)	651 (27.4%)	605 (32.5%)
Medicaid coverage				
Medicaid 1 month before Dx	24 (0.2%)	19 (0.2%)	1964 (82.7%)	1579 (84.8%)
Medicaid 2 months before Dx	36 (0.3%)	19 (0.2%)	1805 (76.0%)	1473 (79.1%)
Individual characteristics				
Male	8572 (71.0%)	6525 (69.0%)	1520 (64.0%)	1190 (63.9%)
non‐Hispanic White	10,153 (84.1%)	7035 (74.4%)	1435 (60.4%)	924 (49.6%)
non‐Hispanic Black	1171 (9.7%)	407 (4.3%)	741 (31.2%)	236 (12.7%)
Hispanic	435 (3.6%)	927 (9.8%)	131 (5.5%)	372 (20.0%)
Married	6870 (56.9%)	5475 (57.9%)	520 (21.9%)	579 (31.1%)
Community characteristics				
Metro	9755 (80.8%)	9116 (96.4%)	1672 (70.4%)	1784 (95.8%)
Census‐tract SES level 1 (poorest)	2028 (16.8%)	1059 (11.2%)	995 (41.9%)	570 (30.6%)
Census‐tract SES level 2	2378 (19.7%)	1626 (17.2%)	565 (23.8%)	482 (25.9%)
Census‐tract SES level 3	2439 (20.2%)	1986 (21.0%)	380 (16.0%)	369 (19.8%)
Census‐tract SES level 4	2656 (22.0%)	2279 (24.1%)	290 (12.2%)	287 (15.4%)
Census‐tract SES level 5 (wealthiest)	2559 (21.2%)	2506 (26.5%)	145 (6.1%)	155 (8.3%)
Tumor characteristics				
Lip site	724 (6.0%)	756 (8.0%)	97 (4.1%)	89 (4.8%)
Tongue site	3537 (29.3%)	2846 (30.1%)	575 (24.2%)	484 (26.0%)
Salivary gland site	1461 (12.1%)	1201 (12.7%)	192 (8.1%)	171 (9.2%)
Mouth floor site	688 (5.7%)	463 (4.9%)	185 (7.8%)	112 (6.0%)
Gum site	1763 (14.6%)	1144 (12.1%)	373 (15.7%)	259 (13.9%)
Nasopharynx site	555 (4.6%)	681 (7.2%)	135 (5.7%)	186 (10.0%)
Tonsil site	1944 (16.1%)	1485 (15.7%)	368 (15.5%)	263 (14.1%)
Oropharynx site	435 (3.6%)	284 (3.0%)	152 (6.4%)	89 (4.8%)
Hypopharynx site	749 (6.2%)	444 (4.7%)	228 (9.6%)	147 (7.9%)
Other oral cavity site	217 (1.8%)	161 (1.7%)	69 (2.9%)	60 (3.2%)
Squamous cell carcinoma histology	9948 (82.4%)	7631 (80.7%)	2024 (85.2%)	1471 (79.0%)
Non‐Oropharyngeal group	6918 (57.3%)	5371 (56.8%)	1299 (54.7%)	1005 (54.0%)
*N*‐sample	12,073	9456	2375	1862
m‐states	8	1	8	1

*Note*: The sample statistics for oral cancer cases in the SEER‐Medicaid data file during the pretreatment period of the study. During this time, no states changed the generosity of their Medicaid dental benefits. The table serves as a baseline measure for outcomes, Medicaid enrollment, tumor‐status, and control variables. *N* = number of observations. % indicates proportion of column. Not California = (Connecticut, Georgia, Iowa, Kentucky, Louisiana, Michigan, New Jersey, and New Mexico).

### 3.1 Association between dropping Medicaid dental coverage and early‐stage oral cancer diagnoses

In the full sample of oral cancer patients enrolled in Medicaid at the time of diagnosis, dropping Medicaid dental coverage was associated with a statistically significant decline in early‐stage oral cancer diagnoses (Est. –3.35%‐points; *p* = 0.034). The estimates were similar for adults enrolled in Medicaid 1–2 months before diagnosis, and for adults continuously enrolled in Medicaid for three months up to the diagnosis (Table 2). Conversely we found that dropping Medicaid dental coverage had no statistically significant associations with early‐stage diagnoses in patients not enrolled in Medicaid.

**TABLE 2:** Estimated association between dropping Medicaid dental coverage and early‐stage oral cancer diagnoses by Medicaid enrollment and oropharyngeal cancer (OPC) status.

		All	OPC	non‐OPC
Medicaid coverage in month of diagnosis	Est.	−0.0335**	0.0029	−0.0646***
	*p*	0.0340	0.7648	0.0080
	95% CI	−0.0951, −0.0035	−0.0786, 0.0694	−0.1454, −0.0321
Medicaid coverage 1 month before diagnosis	Est.	−0.0278**	0.0098	−0.0582***
	*p*	0.0200	0.5285	0.0020
	95% CI	−0.0842, −0.0117	−0.0751, 0.0693	−0.1108, −0.0457
Medicaid coverage 2 months before diagnosis	Est.	−0.0250**	0.0169	−0.0600**
	*p*	0.0420	0.4505	0.0020
	95% CI	−0.0888, −0.0024	−0.0829, 0.0796	−0.1322, −0.0506
Continuous Medicaid coverage for 3 months at diagnosis	Est.	−0.0267**	0.0223	−0.0678**
	*p*	0.0420	0.3584	0.0200
	95% CI	−0.0944, −0.0022	−0.0757, 0.0904	−0.1322, −0.0506
No Medicaid enrollment	Est.	−0.0158	−0.0159	−0.0156
	*p*	0.4464	0.3544	0.4665
	95% CI	−0.0699, 0.0359	−0.0655, 0.0359	−0.0750, 0.0534

*Note*: The estimate of the linear probability regression model testing for an association between dropping Medicaid dental coverage and the probability of an early‐stage oral cancer diagnosis. Medicaid sample ranges from 7522 to 9596. Total non‐Medicaid sample = 50,013. Two‐sided 95% confidence intervals (95% CI) estimated by wild cluster bootstrap algorithm.

** *p* < 0.05.

*** *p* < 0.01.

**FIGURE 1:**
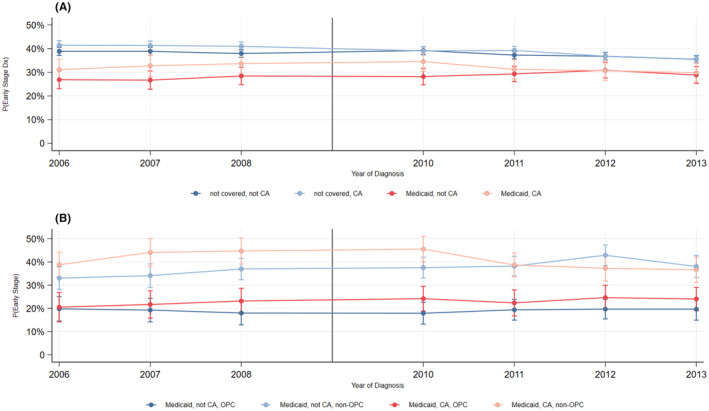
F1 – Year‐by‐year trends in early‐stage oral cancer diagnoses. Panel A plots the trends (%) of early‐stage (0, I, and II) diagnoses of oral and pharyngeal cancer by state (California and not California) and Medicaid enrollment (enrolled at diagnosis and not enrolled at diagnosis). Panel B plots the trends for all Medicaid enrollees with oral and pharyngeal cancers by state (California and not California) and cancer type (OPC = oropharyngeal cancer; non‐OPC = oral cavity cancer). The black vertical line represents 2009, the year California dropped their state Medicaid dental benefits for adults.

When analyzing OPC and non‐OPC separately, we find no effect from dropping Medicaid coverage on early diagnoses of OPC. In contrast, for non‐OPC, we find that dropping Medicaid dental coverage was associated with a statistically significant decline in early‐stage diagnoses (Est. = −6.5%‐point, *p* = 0.008). This decline represented a 20% relative reduction from baseline rates (Figure 1).

Table 3 reports the results of the differential pre‐trend tests. In the full sample and the non‐OPC sample, we found no evidence to suggest that early‐stage diagnosis trends varied significantly between California and other sample states before 2008. However, we found some evidence of differential pre‐trends in the OPC sample. Prior to 2009, the early‐stage OPC diagnosis trends for Medicaid enrollees in California were increasing relative to trends for Medicaid enrollees outside of California. This differential trend may signal an upward bias of the statistically insignificant estimates in the OPC Medicaid sample.

**TABLE 3:** Pretreatment differential trend tests.

	Coef.	All				OPC				non‐OPC			
		Est.	Low CI	High CI	Joint (*p*)	Est.	Low CI	High CI	Joint (*p*)	Est.	Low CI	High CI	Joint (*p*)
Medicaid coverage in month of diagnosis	2006*CA	−0.0157	−0.1595	0.1482	0.875	−0.0409	−0.2174	0.1426	0.927	−0.0001	−0.2278	0.2843	0.957
	2007*CA	−0.0227	−0.1384	0.0896		−0.0452*	−0.1739	0.0563		−0.0069	−0.2042	0.1953	
Medicaid coverage 1 months before diagnosis	2006*CA	−0.0195	−0.1925	0.1486	0.687	−0.0850*	−0.3305	0.1005	0.764	0.0304	−0.1580	0.2909	0.451
	2007*CA	−0.0347	−0.1412	0.0662		−0.0724**	−0.2304	0.0285		−0.0082	−0.1988	0.1843	
Medicaid coverage 2 months before diagnosis	2006*CA	−0.0138	−0.1919	0.1968	0.749	−0.0902*	−0.3166	0.1057	0.981	0.0424	−0.1867	0.3432	0.753
	2007*CA	−0.0300	−0.1292	0.0698		−0.0916**	−0.2630	0.0567		0.0172	−0.1560	0.1880	
Continuous Medicaid coverage for 3 months at diagnosis	2006*CA	−0.0084	−0.1761	0.1832	0.769	−0.0814*	−0.3059	0.1217	0.981	0.0428	−0.1757	0.3471	0.813
	2007*CA	−0.0224	−0.1275	0.0813		−0.0833**	−0.2559	0.0475		0.0232	−0.1751	0.2194	

*Note*: The pretreatment differential trend test between California and other states. Significant differences in the pretreatment would suggest possible violations to the identification assumptions of the study. The table results are based on linear regressions which test for individual coefficient differences by year (2006 or 2007) for California, as well as joint tests (2006 and 2007). Inference is based on traditional clustered standard errors for the coefficient (Est.) and the wild cluster bootstrap (CI and joint *p*‐value).

* *p* < 0.1.

** *p* < 0.05.

### 3.2 Interaction between dropping Medicaid dental coverage and dentists per capita with early‐stage oral cancer diagnoses

Table 4 shows estimates of the interaction effects between dropping Medicaid dental coverage and the county number of dentists per capita. For non‐OPC, dropping dental coverage was associated with a greater decline in likelihood of early diagnosis with more dentists per capita. For patients continuously enrolled for 3 months, dropping dental coverage was associated with a 1.25%‐point decline in early‐stage diagnoses with each additional dentist per 5000 capita (*p* = 0.002). There was no evidence of such an interaction for patients with OPC and for non‐Medicaid patients. There was also no evidence of significant pre‐trends (Table 5).

**TABLE 4:** Estimating the interaction between dropping Medicaid dental coverage and dentists/capita with early‐stage oral cancer diagnoses by Medicaid enrollment and oropharyngeal cancer (OPC) status.

		All	OPC	Non‐OPC
Medicaid coverage in month of diagnosis	Est.	−0.0059**	0.0012	−0.0107***
	*p*	0.0260	0.7107	0.0040
	95% CI	−0.0295, −0.0012	−0.0220, 0.0181	−0.0404, −0.0046
Medicaid coverage 2 months before diagnosis	Est.	−0.0046**	0.0029	−0.0097***
	*p*	0.0380	0.5546	0.0040
	95% CI	−0.0261, −0.0003	−0.0194, 0.0197	−0.0344, −0.0042
Medicaid coverage 3 months before diagnosis	Est.	−0.0042*	0.0051	−0.0113***
	*p*	0.0841	0.4945	0.0040
	95% CI	−0.0296, 0.0006	−0.0227, 0.0242	−0.0390, −0.0056
Continuous Medicaid coverage for 3 months at diagnosis	Est.	−0.0041*	0.0068	−0.0125***
	*p*	0.0881	0.4204	0.0020
	95% CI	−0.0294, 0.0007	−0.0220, 0.0277	−0.0420, −0.0066
No Medicaid enrollment	Est.	−0.0034	−0.0024	−0.0039
	*p*	0.4424	0.3584	0.4705
	95% CI	−0.0196, 0.0102	−0.0187, 0.0104	−0.0210, 0.0154

*Note*: The estimate of the linear probability regression model testing for an association between the interaction of dropping Medicaid dental coverage and dentists per 5000 population with the probability of an early‐stage oral cancer diagnosis. Medicaid sample ranges from 7522 to 9596. Non‐Medicaid sample = 50,013. Two‐sided 95% confidence intervals (95%CI) estimated by wild cluster bootstrap algorithm.

* *p* < 0.1.

** *p* < 0.05.

*** *p* < 0.01.

**TABLE 5:** Pretreatment differential trend tests (interaction between dropping dental coverage and dentists/capita).

	Coef.	All				OPC				Non‐OPC			
		Est.	Low CI	High CI	Joint (*p*)	Est.	Low CI	High CI	Joint (*p*)	Est.	Low CI	High CI	Joint (*p*)
Medicaid coverage in month of diagnosis	2006*CA	−0.0017	−0.0450	0.0379	0.9910	−0.0179	−0.0656	0.0433	0.8869	0.0088	−0.0639	0.0729	0.9469
	2007*CA	−0.0023	−0.0371	0.0197		−0.0162**	−0.0500	0.0174		0.0092	−0.0536	0.0436	
Medicaid coverage 2 months before diagnosis	2006*CA	0.0009	−0.0610	0.0404	0.6126	−0.0235*	−0.0963	0.0380	0.8989	0.0172	−0.0468	0.0705	0.4805
	2007*CA	−0.0048	−0.0406	0.0166		−0.0221***	−0.0651	0.0119		0.0081	−0.0534	0.0427	
Medicaid coverage 3 months before diagnosis	2006*CA	0.0011	−0.0609	0.0507	0.7768	−0.0223*	−0.0888	0.0324	0.9930	0.0165	−0.0535	0.0898	0.9449
	2007*CA	−0.0025	−0.0362	0.0197		−0.0229**	−0.0763	0.0230		0.0130	−0.0394	0.0454	
Continuous Medicaid coverage for 3 months at diagnosis	2006*CA	0.0018	−0.0577	0.0500	0.7447	−0.0201*	−0.0843	0.0381	0.8729	0.0155	−0.0523	0.0868	0.9169
	2007*CA	−0.0022	−0.0359	0.0223		−0.0225**	−0.0713	0.0221		0.0127	−0.0456	0.0509	

*Note*: The pretreatment differential trend test between California and other states. Significant differences in the pretreatment would suggest possible violations to the identification assumptions of the study. The table results are based on linear regressions which test for individual coefficient differences by year (2006 or 2007) for California, as well as joint tests (2006 and 2007). Inference is based on traditional clustered standard errors (Est.) and wild cluster bootstrap (CI and joint *p*‐value).

* *p* < 0.1.

** *p* < 0.05.

*** *p* < 0.01.

## 4 DISCUSSION

This study investigated the effect of dropping Medicaid dental coverage in California on early diagnosis of oral cancers. Using newly released SEER‐Medicaid data that allowed us to identify a cohort of oral cancer patients with verified Medicaid enrollment at the time of and months leading up to diagnosis,^32^ we found that dropping Medicaid dental coverage was associated with lower rates of early‐stage diagnoses of cancers of the oral cavity but not oropharyngeal cancers. The greatest change was found in adults with three continuous months of Medicaid enrollment before diagnosis who lived in areas with more dentists per capita.

Our findings build upon previous work indicating that access to the health system is associated with improved detection outcomes for oral cancer patients.^44^, ^45^, ^46^ Where our study makes its greatest contribution, however, is the explicit emphasis on access to Medicaid dental services. Because dentists provide the bulk of oral cancer screenings, access to dental services promotes earlier oral cancer detection.^5^ However, our findings suggest that access to dental insurance benefits without good access to providers who are willing to accept these insurance benefits may not be sufficient. The results of interactions between dropping dental coverage and dentist supply support this premise; the effect of dropping coverage was larger in areas with more dentists, indicating that patients in these areas stood to lose the most from dropping coverage, likely due to better access (before dropping the coverage) than areas without enough providers. This result is important since adults, specifically low‐income, rural, and racially minoritized populations face additional access barriers to dental care.^15^, ^47^, ^48^ This also highlights the limitations of access to Medicaid dental benefits for improving oral cancer detection in low‐income adults, who reside in areas without adequate providers. This problem is likely even further exacerbated when considering the extra difficulty of finding providers who accept Medicaid dental insurance.^21^

In 2014, California resumed offering dental benefits to their Medicaid enrollees.^17^ This proved to be a significant step toward improving oral health for low‐income California residents. Yet, less is known about how well California had addressed other systemic barriers to accessing dental services during this time. Moreover, since 2009, several other states have changed their Medicaid dental benefits and service delivery. While Medicaid expansion was touted as a critical policy for expanding access to dental services, this benefit was primarily realized in states which both expanded Medicaid under the Affordable Care Act and offered extensive Medicaid dental benefits.^39^, ^49^ During the late 2010s, states also began shifting more of their Medicaid dental service delivery toward privatized, managed care systems.^50^, ^51^ How these changes have impacted access to care for oral cancer remain unclear. Then, in 2020, the pandemic disrupted everything, leading to further delays in care including dental services. Amid rampant volatility, our evidence should motivate further research studying how policies remove or add barriers to dental care, especially as evidence continues to support the critical role of a dental professionals for detecting oral cancer among low‐income adults.

We found that dropping Medicaid dental coverage impacted early diagnosis of cancers of the oral cavity. The location of oral cavity cancers in the mouth may be more visible and detectable by dentists during oral screening. However, we find no evidence that access to Medicaid dental services has affected the detection of oropharyngeal cancer. This result is not surprising, given visual examinations of the mouth during a comprehensive dental exam are less likely to detect abnormalities deep in the head and neck area.^49^, ^52^ Unfortunately, despite the proliferation of a preventive vaccine, OPC incidence is rising.^53^, ^54^ Clearly, improving outcomes for advanced‐stage OPC cannot yet rely on increasing access to dental screening protocols. Rather, policymakers and practitioners must continue implementing and adapting effective HPV‐vaccination programs to prevent OPC across the population.

### 4.1 Strengths and limitations

Among the greatest strength of our study is the analysis of the newly released SEER‐Medicaid data, which, for the first time, provides investigators with valid Medicaid enrollment indicators in the gold‐standard, population‐based cancer registry data file. While the data are novel, however, SEER‐Medicaid only includes nine states. Also, we focus on only one state that dropped dental coverage during the time period available in the data; so the results may only generalize to California. Data limitations and small sample sizes also hindered our ability to conduct subgroup analyses by race, rurality, or other factors (i.e., smoking rates) related to disparate burden of oral cancer outcomes. This study may also be subject to the limitations inherent in any observational study, including potential confounding and selection bias. However, we have attempted to adjust and test for potential threats to internal validity through the difference‐in‐differences design. Finally, we implement a novel inference procedure which reduces our Type 1 error, which elevates the confidence of our findings and implications.

## 5 CONCLUSION

Delayed detection of oral cancer continues to represent a major public health concern and disproportionately impacts low‐income adults. While dentists play a key role in early detection of oral cancer, millions of adults lack access to dental coverage or available providers. Our study contributes new evidence to test how California's decision to drop Medicaid dental benefits in 2009 affected early‐stage oral cancer diagnoses. Among the first to analyze novel SEER‐Medicaid data, this study identified a cohort of oral cancer patients with verified Medicaid enrollment at the time of and months leading to diagnosis. Using a difference‐in‐differences design, we find evidence that dropping Medicaid dental coverage was associated with a 5%–6%‐point decline in the probability of being diagnosed at an early‐stage for Medicaid enrollees with cancer of the oral cavity. The largest declines were found in counties with more dentists per capita. There was no association between dropping Medicaid dental coverage and early diagnosis of oropharyngeal, a finding which warrants alternative policy approaches to address the burden of this growing cancer. Future research is needed to understand how the continued volatility of Medicaid dental coverage and provider shortages may be impacting oral cancer detection.

## AUTHOR CONTRIBUTIONS

**Jason Semprini:** Conceptualization (lead); data curation (lead); formal analysis (lead); funding acquisition (lead); investigation (lead); methodology (lead); project administration (lead); resources (lead); software (lead); supervision (lead); validation (lead); visualization (lead); writing – original draft (lead); writing – review and editing (lead). **Julie Reynolds:** Methodology (supporting); supervision (equal); writing – original draft (equal); writing – review and editing (equal). **Whitney E. Zahnd:** Funding acquisition (supporting); investigation (supporting); methodology (supporting); supervision (equal); writing – original draft (equal); writing – review and editing (equal). **George L. Wehby:** Conceptualization (equal); data curation (equal); formal analysis (equal); funding acquisition (equal); investigation (equal); methodology (equal); project administration (equal); resources (equal); software (equal); supervision (equal); validation (equal); writing – original draft (equal); writing – review and editing (equal).

## FUNDING INFORMATION

National Institute of Dental and Craniofacial Research–1F31DE0032250‐01.

## CONFLICT OF INTEREST STATEMENT

The authors have no conflicts of interest to report.

## ETHICS STATEMENT

This research was determined not to be Human Subjects Research by the University of Iowa Biomedical IRB.

## Data Availability

Data access is subject to third‐party restrictions and is not made available by the authors.
